# Supplementary material for: Bayesian variable selection for genome-wide association study of grain traits in rice
Source: PLoS One. 2026 Mar 17;21(3):e0344021. doi: 10.1371/journal.pone.0344021 (PMC12994784; doi:10.1371/journal.pone.0344021)
Supplement: S1 Text — (PDF) [file pone.0344021.s001.pdf]

Supplementary Materials for "Bayesian Variable Selection for Genome-Wide Association Study of Grain Traits in Rice"

Rupam Basu[1], Sabyasachi Mukhopadhyay[2], and Kaustubh Adhikari[3]
[1]Decision Sciences, IIM Udaipur, Udaipur, India
[2]Operations Management, IIM Calcutta, Kolkata, India
[3]School of Mathematics and Statistics, Open University, London, UK

S1 Data overview and processing

For our analysis, we are using the Rice genotype and phenotype data. The dataset, as described in Section 2 in the main text, consists of observations from 2,266 rice plants, covering 3 distinct traits (phenotypes) and 12,486 genotype markers.

In our analysis, we will use the three phenotypes: **GRLT (Grain length)**, **GRWD (Grain width)**, and **SDHT (Seedling height)** as the response variables. The genotype markers will serve as covariates to explain the phenotypes. The summary of the three variables is shown in Table S1.

Variables	Min	1st Quantile	Mean	Median	3rd Quantile	Max	SD	Variance	Coefficient of variation (CV)
**GRLT**	4.7	8	8.622	8.600	9.300	12.700	1.017	1.034	0.118
**SDHT**	12	32	38.702	38.702	45.000	74.000	10.670	113.856	0.276
**GRWD**	1.5	2.7	3.018	3.000	3.300	4.400	0.411	0.169	0.136

Table S1: Summary statistics **GRLT (Grain length)**, **GRWD (Grain width)**, and **SDHT (Seedling height)**

S1.1 Check for normality assumption of the phenotypes

It is essential to assume the normality of the phenotypes under study to apply a multiple linear regression model or any Bayesian approach mentioned above. To verify this assumption, we first standardized the phenotypes (y) as follows:

$$y_{\text{standardized}} = \frac{y - \bar{y}}{\text{SD}(y)}$$

where $\bar{y}$ is the sample mean and $\text{SD}(y)$ is the standard deviation of the phenotypes. Next, we visualized the standardized phenotypes using a histogram, a Q-Q plot, and a normal quantile line, presented in Figure S1, Figure S2, and Figure S3, respectively.

Additionally, we conducted a formal test for normality using the Jarque–Bera test [Jarque and Bera, 1980]. This test evaluates the goodness-of-fit of the sample data to a normal distribution. The test statistic and corresponding p-values are summarized in Table S2. The Jarque–Bera test statistic (JB) is calculated as follows:

$$JB = \frac{n}{6}\left(S^2 + \frac{(K-3)^2}{4}\right)$$

where n is the sample size, S is the skewness of the distribution, and K is the kurtosis. The hypotheses for the Jarque–Bera test are as follows:

$$H_0 : \text{The data is normally distributed}$$
$$H_1 : \text{The data is not normally distributed}$$

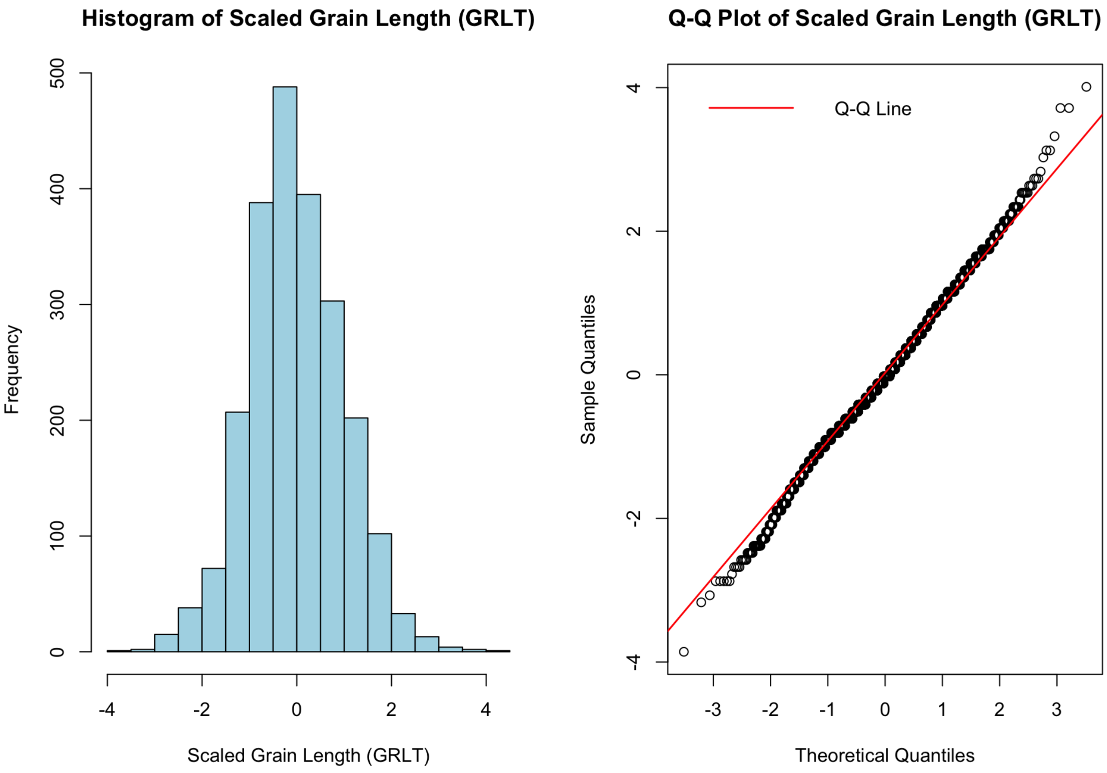

Figure S1: Histogram and Q-Q plot of Scaled Grain Width (GRWD)

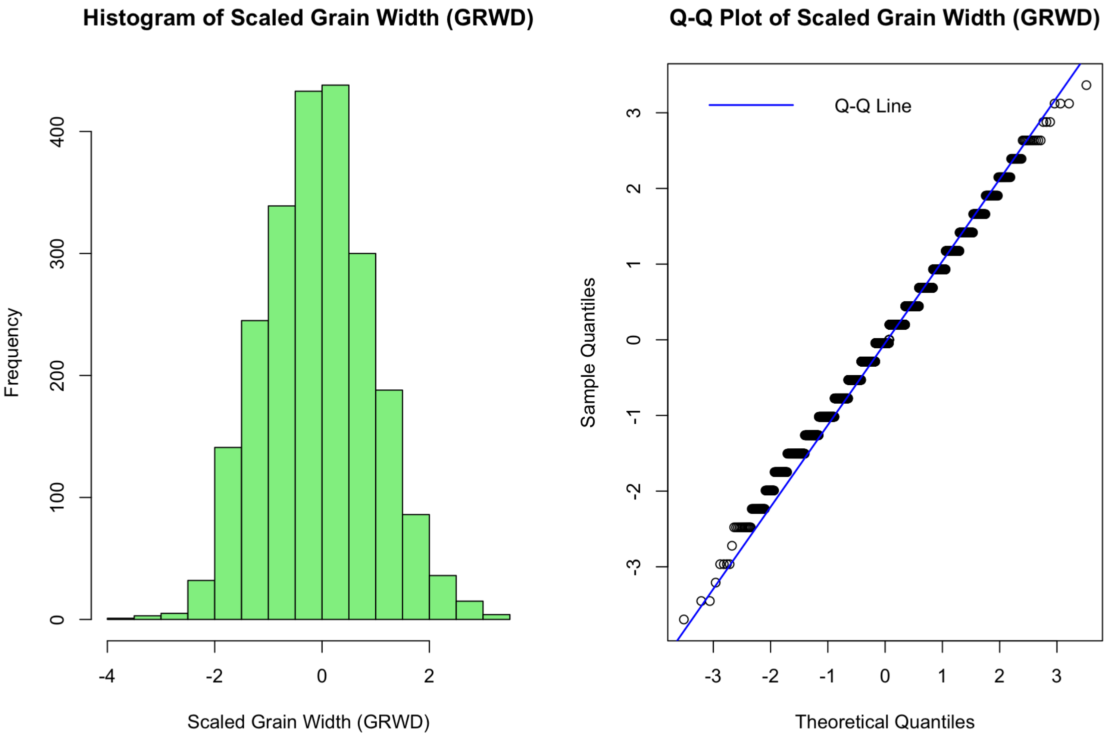

Figure S2: Histogram and Q-Q plot of Scaled Grain Length (GRLT)

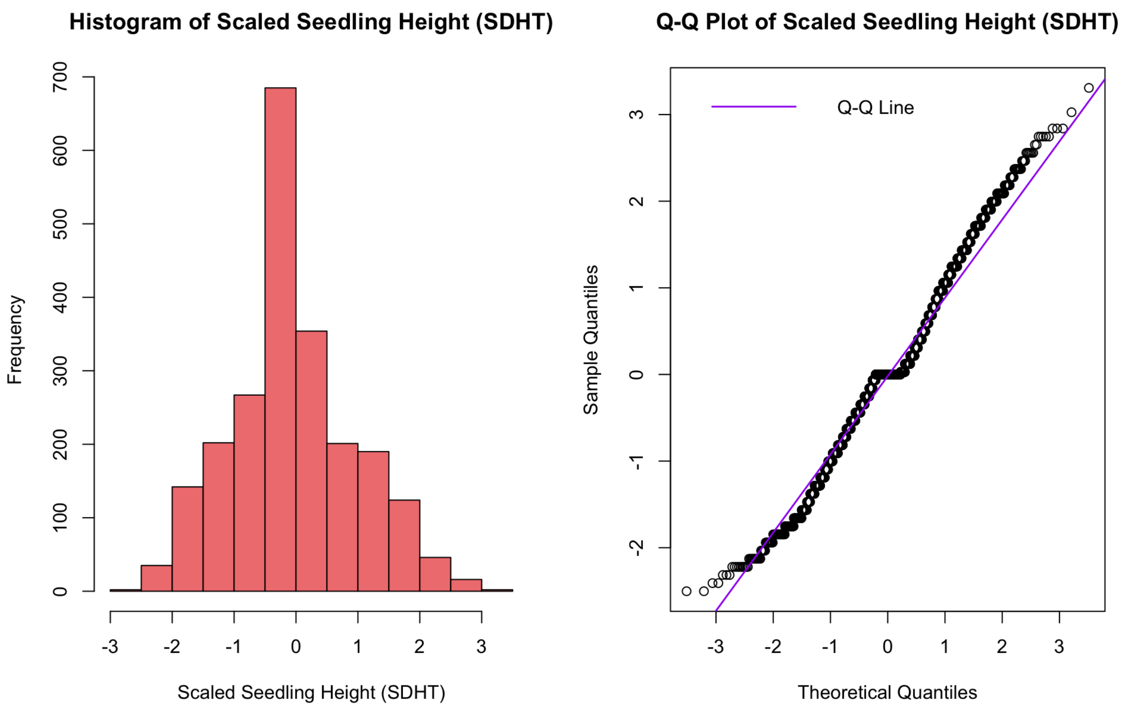

Figure S3: Histogram and Q-Q plot of Scaled Seedling Height (SDHT)

The test statistic follows a chi-squared distribution with two degrees of freedom. If the test statistic exceeds the critical value at a given significance level (α), we reject the null hypothesis H_0 and conclude that the data does not follow a normal distribution.

The corresponding test results, including the test statistic and p-values, are reported in Table S2. These values allow us to evaluate the normality assumption based on the decision criteria. We use the `jarqueberaTest` function from `bestNormalize` [Peterson, 2021] package in R software to get the corresponding test statistics values and the p-values.

**Phenotypes**	JB	**p-value**	**Decision (at 5% significance level)**
**GRLT (Grain length)**	12.9088	0.001574	Reject H_0
**GRWD (Grain width)**	2.2179	0.3299	Accept H_0
**SDHT (Seedling Height)**	12.7862	0.001673	Reject H_0

Table S2: The test statistic JB and $p-values$ without applying transformation

As shown in Figures S1, S2, and S3 and supported by the results in Table S2, the phenotypes GRLT (Grain Length) and SDHT (Seedling Height) exhibit deviations from normality. This is indicated by the shapes of the histograms and the patterns in the Q-Q plots, where both phenotypes show significant departures from the theoretical normal distribution. Additionally, the p-values from the Jarque-Bera tests for these two phenotypes are less than 0.05, leading to the rejection of the normality assumption.

In contrast, the phenotype GRWD (Grain Width) adheres to normality, as evidenced by its Jarque-Bera test, which does not reject the normality assumption ($p-value > 0.05$). This conclusion is further supported by the corresponding histogram and Q-Q plot, which align well with the expected normal distribution.

To achieve the normality assumption for **GRLT** and **SDHT**, we applied *Order Quantile Normalization (OQN)* [Peterson and Cavanaugh, 2020] to these phenotypes. OQN transforms data to follow a normal distribution by mapping quantiles of the original data to the quantiles of a standard normal distribution. Mathematically, this can be expressed as:

$$y_i = \Phi^{-1}\left(\frac{r_i - 0.5}{n}\right)$$

Where y_i is the transformed value, r_i is the rank of the original value, and Φ^{-1} is the inverse

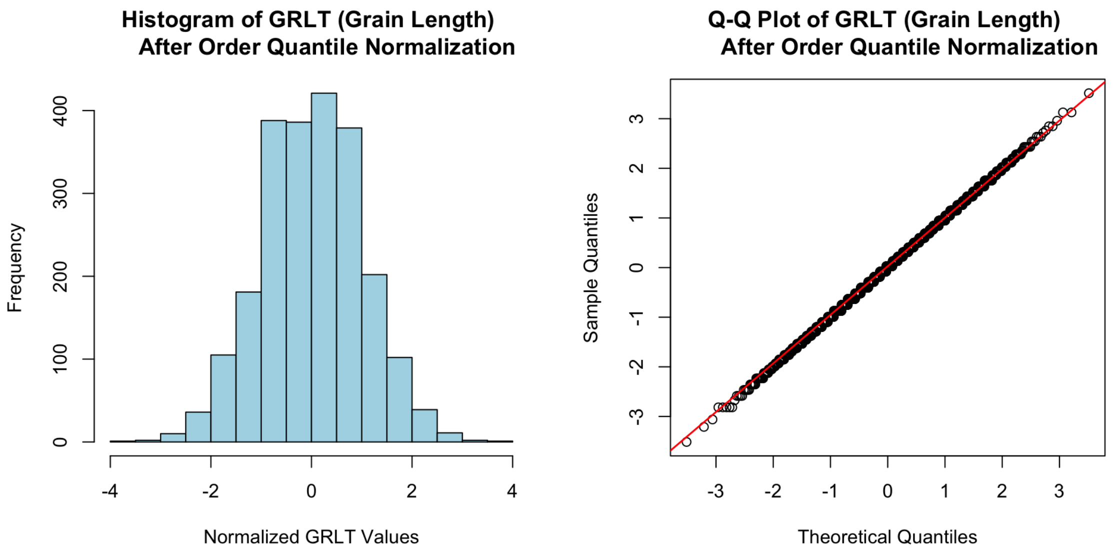

Figure S4: Histogram and Q-Q plot of GRLT (Grain Length) After Order Quantile Normalization

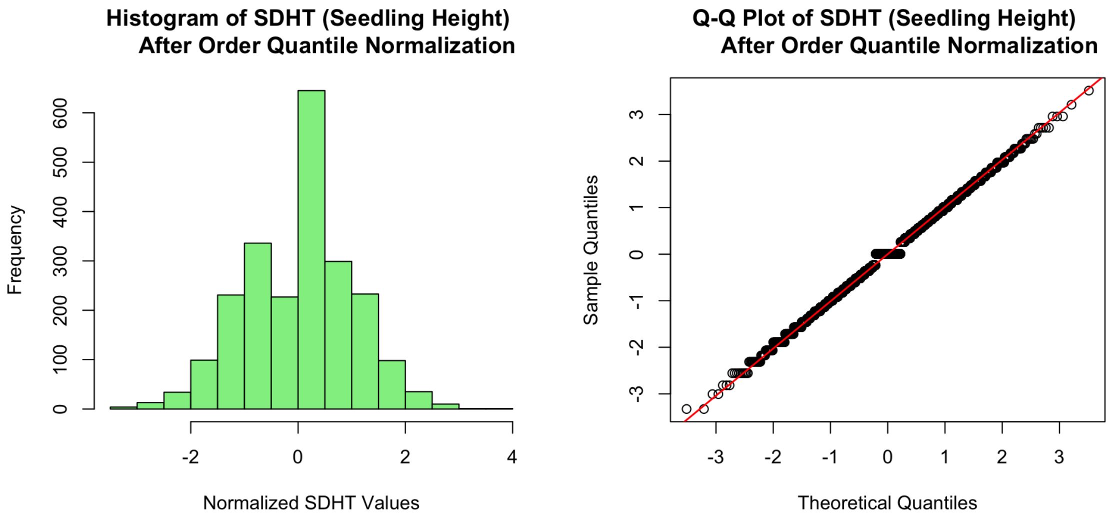

Figure S5: Histogram and Q-Q plot of SDHT (Seedling Height) After Order Quantile Normalization

cumulative distribution function of the standard normal distribution.

We identified the optimal transformation using the `bestNormalize` function from the `bestNormalize` package (Peterson [2021]) in `R`. This function utilizes the *Pearson P-test statistic* to assess normality, selecting the transformation that maximizes the test statistic value. The Pearson P-test statistic is calculated as follows:

$$P = \sum \left(\frac{(O_i - E_i)^2}{E_i} \right)$$

where O_i represents the observed frequency in each class, and E_i represents the expected frequency under the normal distribution. The transformation with the highest Pearson P-test statistic value is considered the best.

After comparing several transformations, we found that order quantile normalization was the most effective for both **GRLT** and **SDHT**. We apply the Order Quantile Normalization (OQN) using the `orderNorm` function from the `bestNormalize` package [Peterson, 2021] in `R` to get the transformed variables. Following the transformation, we generated histograms and Q-Q plots (Figures S4 and S5) and applied the *Jarque-Bera test* (Table S3) to validate the normality assumption. The results indicated that normality holds for both variables after the transformation.

Phenotypes	JB	p-value	Decision (at 5% significance level)
**GRLT (Grain length)**	0.0234	0.9884	Accept H_0
**SDHT (Seedling Height)**	0.0147	0.9927	Accept H_0

Table S3: The JB test statistic and p-values of the phenotypes after applying the transformation

References

Carlos M. Jarque and Anil K. Bera. Efficient tests for normality, homoscedasticity and serial independence of regression residuals. *Economics Letters*, 6(3):255–259, 1980. ISSN 0165-1765. doi: https://doi.org/10.1016/0165-1765(80)90024-5. URL https://www.sciencedirect.com/science/article/pii/0165176580900245.

Ryan A. Peterson. Finding Optimal Normalizing Transformations via bestNormalize. *The R Journal*, 13(1):310–329, 2021. doi: 10.32614/RJ-2021-041.

Ryan A. Peterson and Joseph E. Cavanaugh. Ordered quantile normalization: a semiparametric transformation built for the cross-validation era. *Journal of Applied Statistics*, 47(13-15):2312–2327, 2020. doi: 10.1080/02664763.2019.1630372.
