# Supplementary material for: Bayesian variable selection for genome-wide association study of grain traits in rice
Source: PLoS One. 2026 Mar 17;21(3):e0344021. doi: 10.1371/journal.pone.0344021 (PMC12994784; doi:10.1371/journal.pone.0344021)
Supplement: S2 Text — (PDF) [file pone.0344021.s002.pdf]

Supplementary Materials for "Bayesian Variable Selection for Genome-Wide Association Study of Grain Traits in Rice"

Rupam Basu[1], Sabyasachi Mukhopadhyay[2], and Kaustubh Adhikari[3]
[1]Decision Sciences, IIM Udaipur, Udaipur, India
[2]Operations Management, IIM Calcutta, Kolkata, India
[3]School of Mathematics and Statistics, Open University, London, UK

S2 Gibbs Sampling for Simulating from Posterior Distributions

One of the most common MCMC techniques in genetic models is **Gibbs sampling**. Gibbs sampling is beneficial when the complete conditional distributions of the parameters are easy to sample from. In genetic studies, where $n \ll p$, Gibbs sampling is an efficient method for sampling from high-dimensional posterior distributions.

The Gibbs sampling algorithm works by iteratively sampling each parameter from its full conditional distribution, given the current values of the other parameters. For example, for a parameter vector $\theta = (\theta_1, \theta_2, \ldots, \theta_k)$, Gibbs sampling involves iterating the following steps:

1. Sample θ_1 from $P(\theta_1|\theta_2, \theta_3, \ldots, \theta_k)$.
2. Sample θ_2 from $P(\theta_2|\theta_1, \theta_3, \ldots, \theta_k)$.
3. Continue this process for all parameters $\theta_3, \ldots, \theta_k$.

After several iterations, the algorithm converges to the joint posterior distribution of θ. For high-dimensional Bayesian models like the spike and slab or Bayesian LASSO, Gibbs sampling can efficiently produce samples from the posterior distribution of the SNP effects β_j, given the prior structure of the model.

S2.1 MCMC Implementation in R

For practical implementation, we rely on MCMC techniques using various functions from established R packages. Below are the key packages and functions used for posterior sampling and computation of posterior distributions for different models:

- **Spike and Slab Model**:
  - We use the `lm.spike` function from the `BoomSpikeSlab` package (Scott [2023]) in R to generate posterior samples and compute the posterior distribution of the regression coefficients β_j. This function implements spike and slab priors, allowing us to obtain posterior samples for both large and small SNP effects.

```
library(BoomSpikeSlab)
result <- lm.spike(y ~ ., data = df, niter = 1000)
posterior_samples <- result$beta
```

- **Bayesian LASSO**:
  - For Bayesian LASSO, we use the `ibrm` function from the `hibayes` package (Yin et al. [2024]) in R with the option `method = "BayesL"`. This function implements the Bayesian LASSO with a Laplace prior to the SNP effects, and it generates posterior samples using MCMC.

```
library(hibayes)
result <- ibrm(y ~ 1, data = phenotype, M = genotype,
                  method = "BayesL", niter = 1000, thin = 1,
                  nburn = 100)
posterior_samples <- result$MCMCsamples[["alpha"]]
```

- **Bayesian Sparse Linear Mixed Model (BSLMM)**:
  - For BSLMM, we also use the `ibrm` function from the `hibayes` package (Yin et al. [2024]) with the option `method = "BSLMM"`. This function fits the Bayesian Sparse Linear Mixed Model by combining sparse regression and mixed effects modeling, and it returns posterior samples of both fixed effects (large SNP effects) and random effects (polygenic background effects).

```
result <- ibrm(y ~ 1, data = phenotype, M = genotype,
                  method = "BSLMM", niter = 1000, thin = 1,
                  nburn = 100)
posterior_samples <- result$MCMCsamples[["alpha"]]
```

These R functions handle the posterior sampling through efficient MCMC techniques, allowing us to explore the posterior distributions of β_j and other model parameters. The results from these functions can then be used to estimate posterior means, credible intervals, and other relevant summaries for SNP effects. We have run the Gibbs sampling for each Bayesian models for 25000 iterations and with a burn-in of 5000 and get the posterior sample. We run this process for the three phenotypes grain length(**GRLT**), grain width (**GRWT**), and seedling height (**SDHT**).

References

Steven L. Scott. Mcmc for spike and slab regression, 2023. URL https://cran.r-project.org/package=BoomSpikeSlab. CRAN Repository.

Lilin Yin, Haohao Zhang, and Xiaolei Liu. Individual-level, summary-level and single-step bayesian regression model, 2024. URL https://github.com/YinLiLin/hibayes.
