# Supplementary material for: Bayesian variable selection for genome-wide association study of grain traits in rice
Source: PLoS One. 2026 Mar 17;21(3):e0344021. doi: 10.1371/journal.pone.0344021 (PMC12994784; doi:10.1371/journal.pone.0344021)
Supplement: S3 Text — (PDF) [file pone.0344021.s003.pdf]

Supplementary Materials for "Bayesian Variable Selection for Genome-Wide Association Study of Grain Traits in Rice"

Rupam Basu[1], Sabyasachi Mukhopadhyay[2], and Kaustubh Adhikari[3]
[1]Decision Sciences, IIM Udaipur, Udaipur, India
[2]Operations Management, IIM Calcutta, Kolkata, India
[3]School of Mathematics and Statistics, Open University, London, UK

S3 MCMC Diagnostic Plots

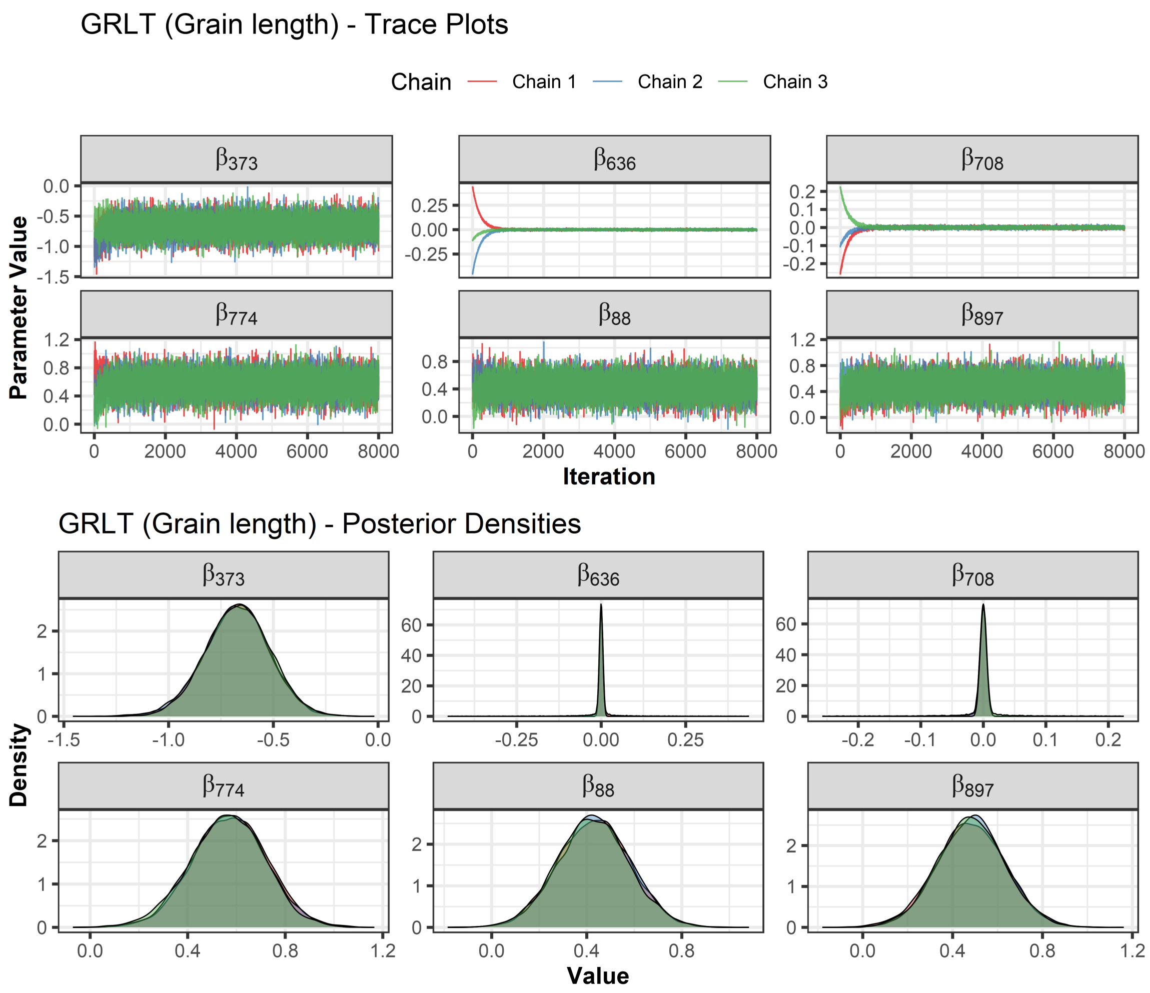

Figure S1: **MCMC convergence diagnostics for the Grain Length (GRLT) phenotype.** Trace plots and posterior density estimates for a random subset of regression coefficients. The trace plots (upper panels) confirm that the three independent chains have mixed well, with no discernible drift or autocorrelation issues. The posterior densities (lower panels) illustrate the shrinkage property of the model: variables with negligible association show sharp posterior accumulation at zero (low dispersion), while significant variables retain wider distributions away from zero.

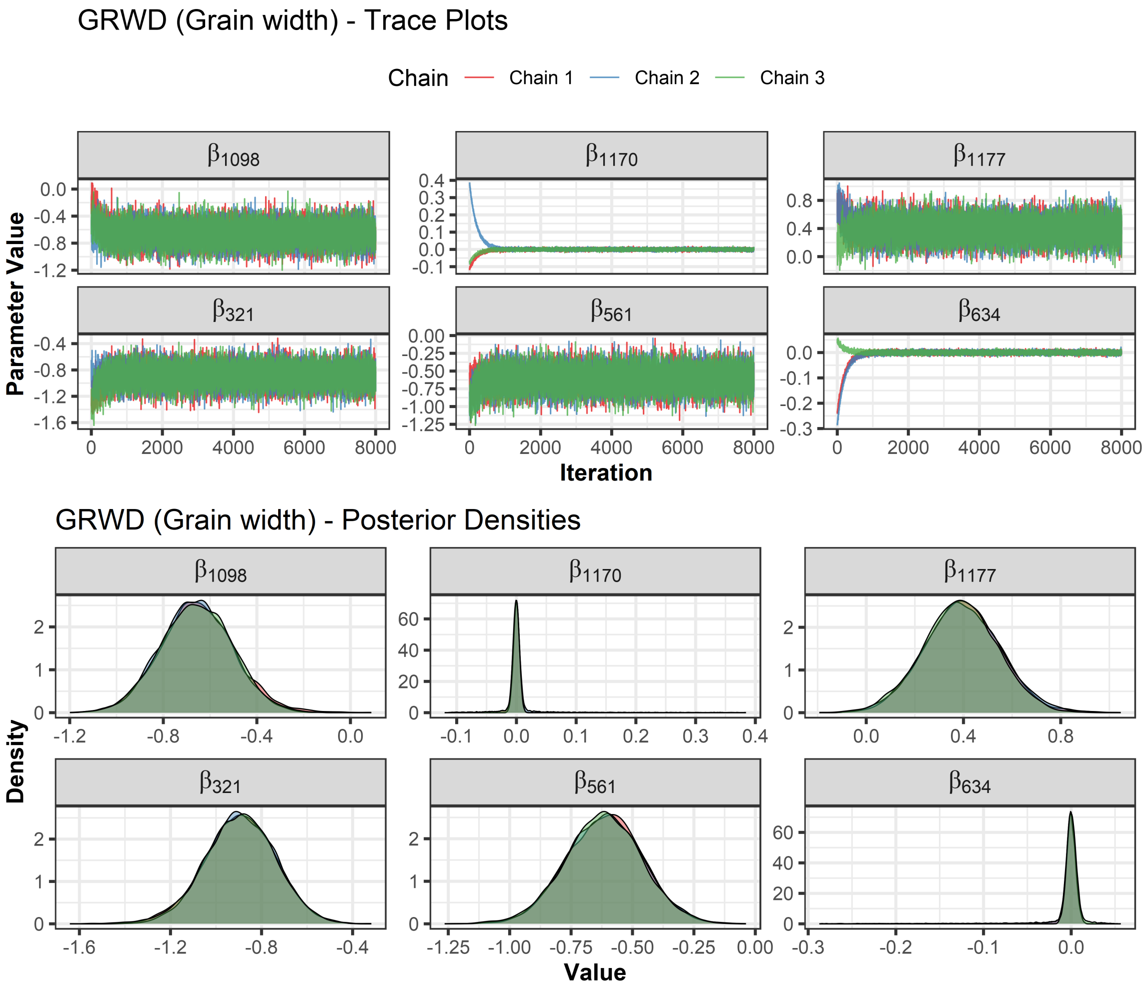

Figure S2: **MCMC convergence diagnostics for the Grain Width (GRWD) phenotype.** Visual assessment of convergence for six representative regression coefficients (β) from the Spike-and-Slab model. **(Top Panels)** Trace plots for three independent MCMC chains (colored by chain) showing stable trajectories and rapid mixing, indicative of convergence to the stationary distribution. **(Bottom Panels)** Corresponding posterior density estimates. Note the distinction in dispersion: coefficients identified as null effects (the "spike" component) exhibit high-density, low-variance distributions centered strictly at zero, whereas active predictors (the "slab" component) display broader posterior support consistent with non-zero effects.

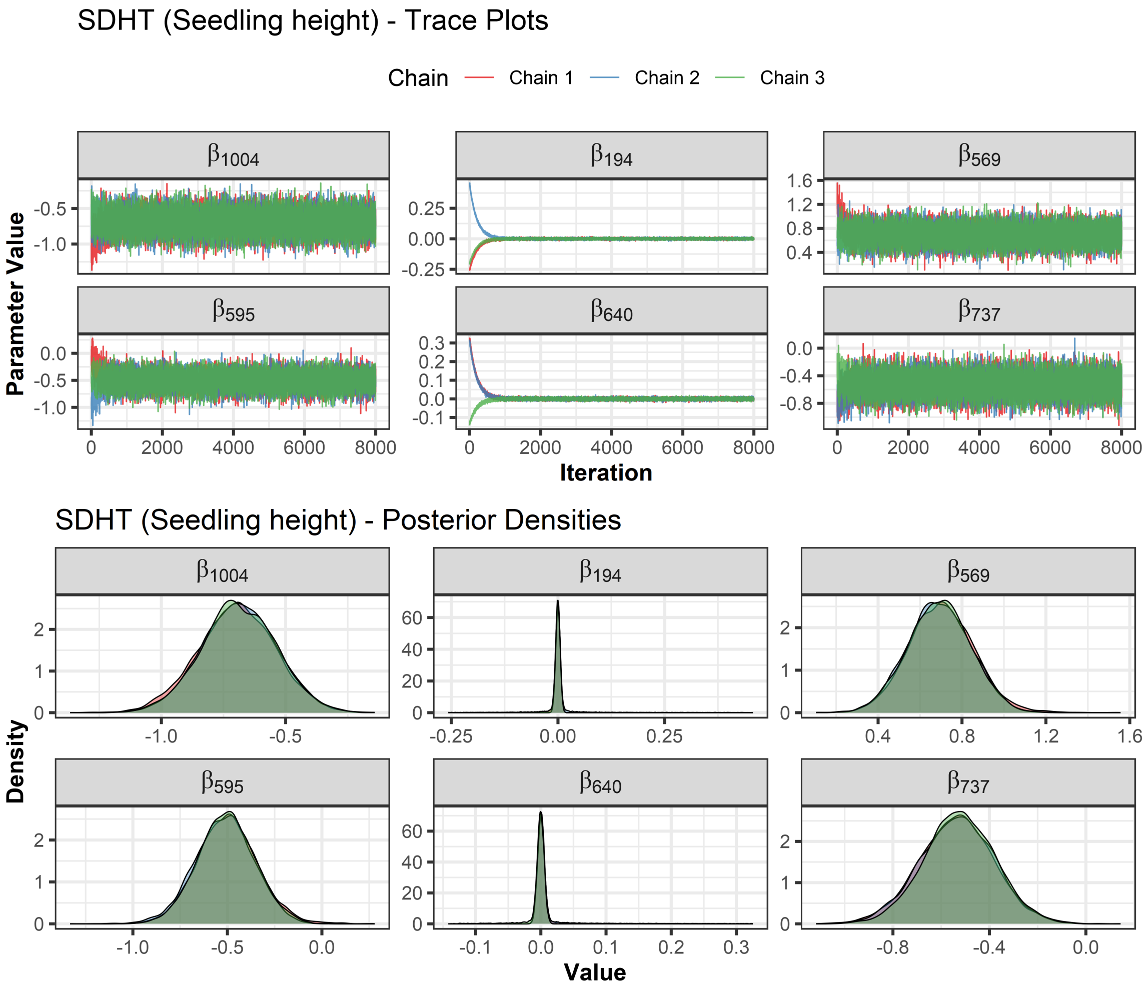

Figure S3: **MCMC convergence diagnostics for the Seedling Height (SDHT) phenotype.** Representative diagnostic plots for the regression coefficients. **A.** Trace plots illustrating the iteration history of the samples across three chains. The overlapping trajectories indicate that the chains have forgotten their initial starting values and converged to a common posterior. **B.** Kernel density estimates of the posterior distributions. The visual evidence supports the model's ability to discriminate between sparse signals (narrow peaks at $\beta \approx 0$) and active genetic effects (dispersed distributions), confirming the effective implementation of the variable selection prior.
